# Analysis of the Spatial Organization of Pastures as a Contact Network, Implications for Potential Disease Spread and Biosecurity in Livestock, France, 2010

**DOI:** 10.1371/journal.pone.0169881

**Published:** 2017-01-06

**Authors:** Aurore Palisson, Aurélie Courcoul, Benoit Durand

**Affiliations:** 1 University Paris Sud, Orsay, France; 2 University Paris Est, Anses, Laboratory for Animal Health, Epidemiology Unit, Maisons-Alfort, France; Centre de physique theorique, FRANCE

## Abstract

The use of pastures is part of common herd management practices for livestock animals, but contagion between animals located on neighbouring pastures is one of the major modes of infectious disease transmission between herds. At the population level, this transmission is strongly constrained by the spatial organization of pastures. The aim of this study was to answer two questions: (i) is the spatial configuration of pastures favourable to the spread of infectious diseases in France? (ii) would biosecurity measures allow decreasing this vulnerability? Based on GIS data, the spatial organization of pastures was represented using networks. Nodes were the 3,159,787 pastures reported in 2010 by the French breeders to claim the Common Agricultural Policy subsidies. Links connected pastures when the distance between them was below a predefined threshold. Premises networks were obtained by aggregating into a single node all the pastures under the same ownership. Although the pastures network was very fragmented when the distance threshold was short (1.5 meters, relevant for a directly-transmitted disease), it was not the case when the distance threshold was larger (500 m, relevant for a vector-borne disease: 97% of the nodes in the largest connected component). The premises network was highly connected as the largest connected component always included more than 83% of the nodes, whatever the distance threshold. Percolation analyses were performed to model the population-level efficacy of biosecurity measures. Percolation thresholds varied according to the modelled biosecurity measures and to the distance threshold. They were globally high (e.g. >17% of nodes had to be removed, mimicking the confinement of animals inside farm buildings, to obtain the disappearance of the large connected component). The network of pastures thus appeared vulnerable to the spread of diseases in France. Only a large acceptance of biosecurity measures by breeders would allow reducing this structural risk.

## 1. Introduction

The use of pastures, defined here as fenced fields used for grazing, is part of common herd management practices for cattle, sheep and other livestock animals. However, for a number of pathogens, zoonotic (e.g. bovine tuberculosis, Rift Valley fever) or not (e.g. foot-and-mouth disease, bluetongue), pastures are places where animals may get infected, allowing the disease to spread from one herd to another one. Depending on the pathogen, grazing animals may acquire infection through two mechanisms. Firstly, direct contacts (nose-to-nose contacts) over the fences with infected animals of a neighbouring infected herd may allow disease transmission. This transmission mechanism is well established for diseases such as bovine viral diarrhoea (BVD) [1,2] or bovine tuberculosis (bTB) [3,4]. Secondly, infection can occur through indirect contacts. For example, airborne transmission has been demonstrated for several diseases like Q-Fever [5]. When an infected animal located on a given pastures excretes the pathogen, all the animals located downwind are exposed to infection. Another example is vector-borne transmission. A blood-sucking insect (e.g. a *Culicoides* or *Culex*) bites an infected grazing animal and later transmits the infection to another animal during a blood meal. This is the major transmission route for pathogens such as bluetongue virus [6], Schmallenberg virus [7] or Rift valley fever virus [8]. In 2006, the bluetongue virus of serotype 8 (BTV-8) was introduced in Belgium, close to the borders with Germany and the Netherlands, and quickly spread in these 3 countries [9]. By the end of 2009, BTV-8 had spread to most countries in western and central Europe, including the United Kingdom, Denmark, Norway, Sweden, Czech Republic, Hungary, Austria, Italy, Luxembourg, Spain, and France [10–13]. This example clearly shows the ability of a vector-borne disease to form an epidemic wave that travels at a continental scale, and the velocity of this wave has been related to the local density of pastures [14], because *Culicoides* vectors can only fly over relatively small distances (< 3 km) [15]. More generally, whatever the transmission mechanism (direct, indirect, vector-borne), the spatial organization of pastures strongly constraints the risk of contagion between animals located on these pastures [16,17]. At the pastures level, this risk appears higher in areas where pastures are adjacent than in areas where they are fragmented and separated by arable land or by natural areas. Similarly, at the herd level, this risk appears higher for herds with many small pastures spread over a large area than for herds with only a few large pastures located in the same area.

The aim of biosecurity measures is to decrease the risk of introduction and spread of disease agents. Saegerman *et al*. [18] classified biosecurity measures in five classes according to the aim of the measures: (i) to limit the risk of introduction (bio-exclusion), (ii) to limit the spread of the pathogen within the same facility (bio-compartmentation), (iii) to limit the spread of the disease agent outside the facility (bio-containment), (iv) to prevent the risk of human bio-contamination and (v) to prevent any environmental bio-contamination and persistence of the pathogen. Three types of measures were considered here (**Table 1**):

- “Strict biosecurity” measures (bio-compartmentation and bio-containment): they are focused on the transmission risk between a pasture and any other pasture. If the pathogen reaches a pasture (and if biosecurity measures are effective), it cannot infect the animals grazing on the neighbouring pastures (pastures of the same or other premises). An example of such biosecurity would consist in confining animals within buildings or applying between-premises biosecurity for all the neighbouring premises.
- “Within-premises biosecurity” measures (bio-compartmentation): they are focused on the transmission risk between pastures of the same premises. If the pathogen reaches a pasture, it cannot infect the animals located on the other pastures of the same premises. An example of such biosecurity would consist in avoiding movements of animals between pastures of the same herd.
- “Between-premises biosecurity” measures (bio-containment): they are focused on the transmission risk between pastures of a give pair of premises. If the pathogen reaches a pasture of the first premises, it cannot infect the animals grazing on the pastures of the second premises. An example of such biosecurity would consist in strengthening the fences (e.g. by installing double fences) between pastures of the two premises.

**Table 1:** Examples of short-term and long-term biosecurity measures to prevent disease transmission on pastures between animals of the same premises (within-premises biosecurity), between animals of different premises (between-premises biosecurity), or both (strict biosecurity).

	Focus	Short-term measures	Long-term measures	Network model
Strict biosecurity	Transmission risk between a pasture and any other pasture	Confining animals inside buildings	Grazing animals on pastures without neighbouring pastures, strengthening of fences or replanting hedges around all the pastures of a premises	Node removal procedure
Within-premises biosecurity	Transmission risk between pastures of the same premises	Standstill of animal movements between pastures	Using a single pasture for each animal batch	Node transformation procedure
Between-premises biosecurity	Transmission risk between pastures of premises A and B	Grazing animals from A on pastures without neighbouring pastures belonging to B	Strengthening of fences, replanting hedges between pastures of A and B	Link removal procedure

Of course, the relevance of these biosecurity measures varies with the disease, and their acceptability by breeders depends on whether they are implemented as short-term (emergency measures for the control of an epidemic) or long-term strategies (changes of herd management practices, land use planning) (**Table 1**). However, even if they are assumed 100% effective at a local scale (e.g. if double fences completely prevent pathogen transmission for a directly-transmitted disease), we do not know to what extent these measures have to be adopted by breeders to be effective at the population level (e.g. how many double fences need to be installed to prevent disease spread at the country level?).

For a given disease, pathogen transmission between animals located on neighbouring pastures only represents part of the overall transmission risk, which also includes the risk induced by live animal trade, for instance. Furthermore, for disease transmitted by direct contact (like bTB), cattle trade and contacts on pastures may not play the same role: cattle trade may allow long-range spread of the disease whereas contacts on pastures may allow short-range spread of the disease [19]. Here we chose to focus on this “pastures component” of the overall transmission risk. This component represents the potential spread of a disease if all animal trade were controlled [20].

The aim of this study was to analyse the risk of disease transmission between herds through contacts between animals located on neighbouring pastures, and to determine whether this risk may be controlled. This contagion risk was analysed as a structural risk, induced by both the spatial organization of pastures and the functioning of breeding systems. It was also analysed as a generic risk, relevant for various infectious diseases, although the epidemiology of a specific disease impacts the probability for an animal to get infected. Two questions were addressed: (i) is the spatial configuration of pastures favourable to the spread of infectious diseases in France? (ii) if yes, which biosecurity measures would allow decreasing this vulnerability, and to what extent would they need to be adopted by breeders?

A common way to represent the structural risk of infectious disease transmission between premises is through a network in which nodes represent premises and links represent contacts between premises that may allow disease transmission. This approach has been widely applied to the risk of disease transmission between herds by live animal trade. The corresponding networks have been studied using network analysis methods in several countries like France [21,22], Great Britain [23], Denmark [24], Italy [25] or Germany [26]. In such networks, herds are represented by nodes and animal trade between herds by links. Hence, the network analysis allows studying the direct or indirect links between herds, because of animal trade. To describe a network, network indicators, like the degree (i.e. the number of nodes directly connected to the node), the betweenness centrality (i.e. the number of shortest paths between pairs of nodes that go through the node) or the clustering coefficient (i.e. a measure of how the nodes tend to cluster together) are computed, and the presence of a giant strongly connected component in the network (i.e. a subnetwork in which all nodes can reach each other) is checked. The presence of a giant strongly connected components is an indicator of the network vulnerability to the spread of an infectious disease as, if introduced into any node of a giant strongly connected component, the disease may potentially reach all the other nodes [23]. Kao *et al*. [23] and Kiss *et al*. [27] showed that the size of the giant strongly connected component was a good predictor to estimate a potential epidemic size.

To assess the efficacy of measures aiming at controlling the risk of disease spread by live animal trade, percolation analysis on the giant component is often implemented: it aims at breaking up the giant component into disconnected small components. In statistical physics, percolation studies the transition of some porous material, represented by a three-dimensional network, from a permeable state to a non-permeable state, when elementary elements of the network are removed (either nodes in the so-called “site percolation”, or links in the so-called “bond percolation”). Applied to network analysis, percolation analyses how a network may be fragmented into smaller networks when some of its components are removed [28]. The resilience of networks against such removal procedures is known to vary according to network topology. For instance, scale-free networks, like animal trade networks, are known to be resilient against random node (or link) removal, but vulnerable to attacks targeted on the most central nodes [21,23,24,26,29–34].

We applied network analysis methodology to the French network of pastures to analyse, at the country level and for 2010, the structural risk of infectious disease spread between farms related to the spatial organization of their pastures. As far as we know, contrary to what was done on live animal trade networks, this methodology has never been used on pastures networks. Two networks were considered: (i) a pasture network with pastures as nodes and links representing potential contacts between animals located on distinct pastures that may allow disease transmission; and (ii) a premises network with premises as nodes and links obtained by aggregating the pastures network (i.e. a link between two premises exists if at least one link between pastures of both premises exists in the pastures network). Two pastures were defined as “in contact” if the distance between both of them was lower than a given threshold. Small thresholds represented the transmission risk for a directly-transmitted disease, whereas larger thresholds represented the transmission risk for an indirectly-transmitted disease. In the first part of the study, the pastures networks obtained for increasing thresholds were analysed and described (network indicators and topological characteristics). The second part of the study focused on the corresponding premises networks and their vulnerability to infectious disease spread. The three different types of biosecurity measures (strict biosecurity, within-premises biosecurity and between-premises biosecurity) were modelled as network modifications (**Table 1**), and three percolations analyses were performed to assess their efficacy at the population level. The first one consisted of node removal to model strict biosecurity measures (e.g. the confinement of animals inside buildings). The second one was based on the transformation of nodes to model within-premises biosecurity measures (e.g. standstill of animal movements between pastures), and lastly, in the third one, links between pastures of different premises were removed to model between-premises biosecurity measures (e.g. double fencing).

## 2. Materials and Methods

### 2.1. Data

The anonymized Land Registration System (geographic database of parcels used for agriculture) of 2010 was obtained from the “Agence de Services et de Paiement” (ASP). This database collects data provided by farmers to claim the Common Agricultural Policy (CAP) subsidies. A CAP subsidy claim consists in a report made by a farmer specifying the geographic location and border of each land parcel she/he owns, and the usage of this parcel for agriculture (using a specific nomenclature) for a given year. Parts of these reports are controlled by local administrations. The number of such controls per member state is fixed by European legislation. Discrepancies between the control result and the farmer’s report may lead to penal consequences for the farmer. CAP reports allow farmers receiving EU aids, but they are not mandatory. However, although data may not be comprehensive, it can reasonably be assumed that the vast majority of farmers do claim CAP subsidies.

**Fig 1:**
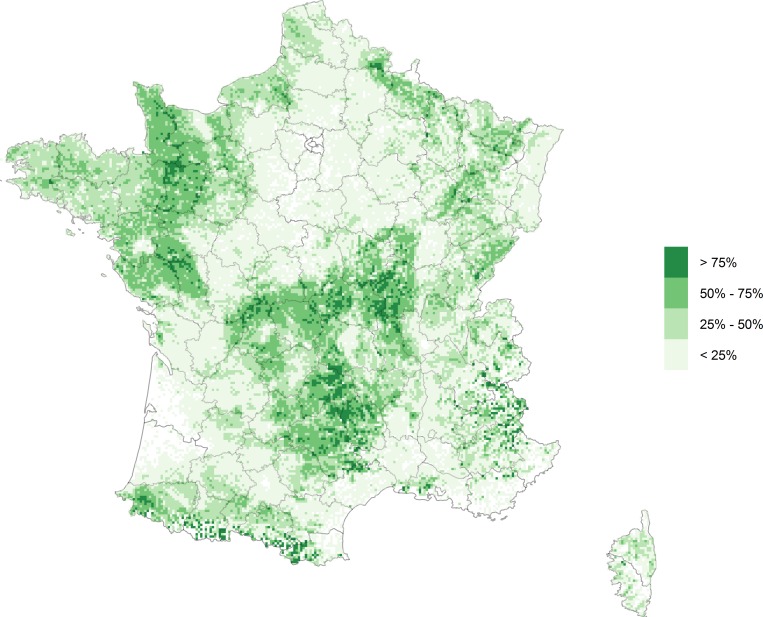
Proportion of land surface covered by pastures. Pastures were aggregated using a square grid (cells of 16km^2^).

We used a spatial layer in which each polygon represents a parcel. The accuracy of these polygons is considered good, with errors below a few meters. Attribute data were the parcel usage reported by the farmer (e.g. crops, grasslands…), the parcel area and an anonymous farmer ID. Among their various usages, parcels dedicated to fodder crops, mountain pastures and permanent or temporary grasslands were considered as “pastures”. The dataset described 6,123,259 parcels, of which 52% (3,159,787) were pastures. These pastures had been reported in 288,066 CAP reports. We assumed that each CAP report corresponded to a given farm, termed below “premises”. Most of the pastures were located in the middle of the country (“Massif central” area). There were also a high density of pastures in the mountains (in the Pyrenees at the Spanish border, in the Alps at the Italian border, in the Jura at the Swiss border and in the Vosges near the German border), near the Belgian border and in the western part of the country (the Brittany, the Normandy and the “Pays de la Loire” regions–**Fig 1**). On average, pastures were small (0.05 km^2^ on average) and two pastures of the same premises could be a couple of kilometres apart. Typical premises owned about ten pastures (**Table 2**).

**Table 2:** Characteristics of the French pastures.

	Mean	Median	2.5th percentile	97.5th percentile
Pastures area (km^*2*^)	0.050	0.0198	0.001	0.259
Number of pastures per premises	11	7	1	42
Area of pastures per premises (km^*2*^)	0.535	0.358	0.009	1.985
Distance between the centroid of a pasture and the centroid of all the pastures of the same premises (km)	2.645	1.221	0.090	13.740

The dataset we used was anonymized, and the presence and type of livestock in premises was unknown. However, several arguments indicate that most of the premises of our dataset corresponded to cattle breeders:

- According to the 2010 agricultural census, 19 million cattle were present in France in 2010, 8 million small ruminants (mainly sheep) and 1 million horses. Cattle thus represent 68% of herbivorous livestock.
- A strong correlation was observed between the number of pastures in the dataset we used and the number of cows per department (source: cattle tracing system database, described in [19]): 0.82 (Spearman’s rank correlation).
- In 2010, the total number of cattle herds housing cows was 232,566 (source: cattle tracing system database) and, according to the 2010 agricultural census, 97% of the farms housing cows did own pastures and used it for breeding. The number of cattle breeders owning cows and pastures can thus be estimated at 225,589 (i.e. 97% of 232,566 farms). This number corresponds to 78% of the 288,066 premises of our dataset. Because the latter figure only concerns farms holding cows, it is a low estimate of the proportion of cattle farms in our dataset (some cattle farmers may own pastures and young cattle but not cows).

The vast majority of the premises (>78%) of our dataset can thus be considered as cattle farms.

In order to build a network of pastures, buffer areas were drawn around parcels. Two pastures were considered “in contact” if their buffer areas intersected. Several buffer widths were used: from 1.5 meters (to model contacts “nose-to-nose over the fence”, while taking into account the accuracy of the database) to 500 meters (to model vector-borne transmission while maintaining a reasonable computation burden). For a given buffer width, a network (termed below “pastures network”) was built with pastures as nodes and links for intersecting buffers. Links represented contacts that may allow disease transmission, between animals located on distinct pastures. A second type of network (termed below “premises network”) was built for each buffer width, by aggregating pastures per premise. These premises networks represented contacts between premises because of contacts between their pastures: nodes were premises and links represented contacts between at least one pasture of the two connected premises. For all networks, links were non-directed since the contact between two pastures was symmetrical. Furthermore, all contacts between two given pastures (pastures network) or between two given premises (premises network) were consolidated in a single link. Animals usually stay several weeks or months on a given pasture; the above networks were thus considered static. Furthermore, they represented a “worst case scenario”, in which all pastures were used at the same time.

### 2.2. Pastures networks analyses and disease implications

Network analysis methods were applied to the pastures networks to assess whether the spatial configuration of pastures was favourable to the spread of infectious diseases. Pastures networks obtained using increasing buffer widths were described using network indicators: (i) the size (the number of nodes), (ii) the number of links, (iii) the size of the largest connected component, (iv) the density (the number of links among all the possible links), (v) the clustering coefficient (the proportion of two connected nodes linked to the same third node), (vi) the average path length, (vii) the assortativity (the correlation between the degrees of linked nodes), (viii) the degree (the number of nodes connected to the node), and (ix) the betweenness centrality (the frequency with which a node is on the shortest path between the different pairs of nodes) [20].

The calculation of the exact values of the average path length and of the betweenness centrality requires generating the full set of shortest paths between any pair of nodes of the network. Due to the size of the networks studied here (>3 million nodes in the pastures network), it was not possible to perform this calculation in a reasonable computation time. We computed instead estimated values of these indicators, based on a random set of 10,000 nodes, using STAN software [35].

To qualify the topology of the pastures networks, we plotted and graphically analysed their degree distributions on a log-log scale. We also compared the values of the clustering coefficient and of the average path length with those obtained in random networks of the same size (same number of nodes and links). Indeed, a linear trend of the degree distribution on a log-log scale is observed in scale-free networks [36]. Furthermore, according to Dubé *et al*. [37], the following two elements are indicative of a small-world topology: (i) a clustering coefficient >20 times greater than that of a random network having the same size, and (ii) similar average path lengths in both networks.

In a pastures network, nodes (i.e. pastures) were connected if they were close enough for their buffer areas to intersect. This type of network thus represented the disease transmission risk assuming no other contact between pastures. However, usual breeding practices involve regular movements of animals by breeders, between the pastures they own, to optimize the use of grasslands. Such movements and, more generally, any indirect contact between the animals grazing on distinct pastures of the same premises allowing disease transmission, may impact the vulnerability of the pastures network. Contacts allowing disease transmission between animals located on non-connected pastures of the same premises were thus modelled by adding the corresponding links, with a probability *p*. For each buffer width, such links were randomly added to the pastures network, using several values of *p*, in order to determine the threshold for which the largest connected component included more than 50% of the nodes. The value of *p* was calculated using a bisection algorithm with a precision of 0.5% (5 realizations of the algorithm to assess the stability of the result).

### 2.3. Premises networks analyses and disease implications

Premises networks obtained with increasing buffer widths were described using network indicators: (i) the number of nodes, (ii) the number of links, (iii) the size of the largest connected component, (iv) the density, (v) the clustering coefficient, (vi) the average path length, (vii) the assortativity, (viii) the degree, and (ix) the betweenness centrality.

As for the pasture networks, to qualify the topology of the premises networks, we plotted and graphically analysed their degree distributions on a log-log scale. We also compared the values of the clustering coefficient and of the average path length with those obtained for random networks of the same size (same number of nodes and links).

Three percolations analyses were then performed to assess the efficacy of the three types of biosecurity measures (**Table 1**). The aim of these percolation analyses was to estimate a threshold below which the largest connected component included less than 50% of the nodes. Each of these percolation analyses was based upon the following general procedure:

1. select the nodes/links with the 1% highest betweenness centrality,
2. apply a change to these nodes/links (a simple removal, or a more complex change that models biosecurity measures),
3. calculate the size of the largest connected component,
4. if this size is <50% of the nodes (i.e. if the giant connected component has disappeared) stop, else go to step (i).

The result of this procedure is the final proportion of nodes/links submitted to changes in step (ii), termed below percolation threshold. It represents the proportion of premises that have to adopt the biosecurity measures (modelled by the changes applied in step (ii)) so that these measures are effective at the population level (i.e. make the network non-vulnerable to the spread of an infectious disease). The higher the percolation threshold was, the more difficult to implement the modelled biosecurity measure was. We chose to target the nodes according to their betweenness centrality (estimated based on shortest paths of length ≤ 3 due to computational time), since it is often the optimal strategy reported in the literature [21,26,29]. We confirmed it was the case for one of the networks studied here by checking that the percolation threshold obtained using the betweenness-based targeted selection was lower than the thresholds obtained using either a degree-based targeted selection or a non-targeted (i.e. random) selection (S1 Appendix). We also checked that the number of nodes removed at each step (i) in the percolation procedure above (the 1% nodes highest betweenness centrality) did not impact the estimate of the percolation threshold (S2 Appendix).

The potential efficacy of the three levels of biosecurity measures presented in **Table 1**was analysed using three percolation procedures differing according to the changes applied to the selected nodes/links (i.e. step (ii) of the general procedure above). To represent strict biosecurity, selected network elements were nodes, which were removed. This removal modelled, for example, the confinement of animals inside buildings. For the within-premises biosecurity, selected network elements were also nodes, but the changes applied to each of them consisted in a subdivision of the node (i.e. the premises) in sub-nodes, each sub-node corresponding to virtual sub-premises having a single pasture. This node subdivision gradually broke up a premises network into a pastures network. It modelled, for example, a standstill of animal movements between pastures of the corresponding premises. For the between-premises biosecurity, selected network elements were links and each of them was removed. This removal modelled, for example, the installation of double fences between pastures of a given pair of premises.

Network analyses were performed using the Igraph package [38] for R 3.1 [39] and the SNAP software [35]. Maps were drawn with the sp package [40] for R 3.1 [39].

## 3. Results

### 3.1. Pastures networks analyses and disease implications

**Fig 2:**
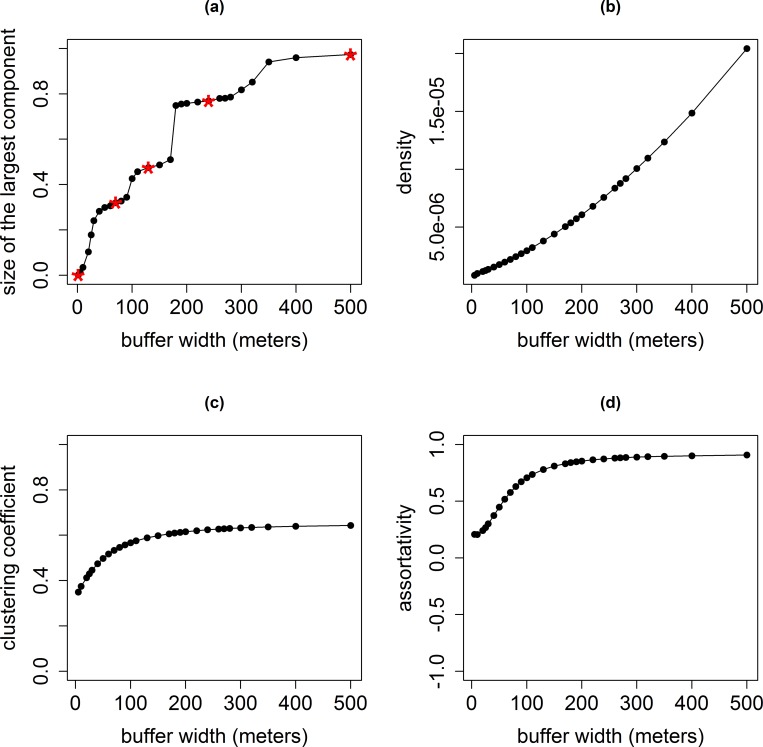
Evolution of network indicators for the pastures network according to the buffer width. (a): proportion of the pastures in the largest connected component (stars: the buffer widths for which network indicators are detailed in **Table 3**and **Table 4**: 1.5, 70, 130, 240 and 500 meters); (b): the density; (c): the clustering coefficient; (d): the assortativity.

The size of the largest connected component was computed for the pastures networks obtained with thirty increasing buffer widths, from 1.5 to 500 meters. As expected, the wider the buffer was, the larger the main connected component was. Four sharp increases were separated by three plateaus: a first plateau with a buffer width between 30 and 100 meters, a second plateau with a buffer width between 100 and 180 meters, and a third plateau with a buffer width between 180 and 300 meters (**Fig 2A**). Consequently, five buffer widths were selected for further analyses: the minimal width (1.5 m), the middle of each plateau (70 m, 130 m and 240 m), and the maximal width (500 m). The pastures network obtained with the smallest buffer width (1.5 meter) was strongly fragmented, with 0.1% of nodes in the largest connected component. This proportion rose to about 50% for a buffer width of 130 meters. The largest connected component included almost all the pastures when the buffer width was 500 meters (Table 3).

**Table 3:** Network indicators describing five pastures networks generated using increasing buffer widths.

Buffer width	1.5 m	70 m	130 m	240 m	500 m
Number of nodes	3,159,787	3,159,787	3,159,787	3,159,787	3,159,787
Number of links	2,438,620	11,049,232	18,978,231	37,794,387	101,984,579
Size of the largest connected component (% of the French pastures)	4,168 (0.1%)	1,011,136 (32%)	1,501,267 (48%)	2,429,220 (77%)	3,079,099 (97%)
Density (x10^-6^)	*	2.21	3.80	7.57	20.43
Clustering coefficient	*	0.53	0.59	0.62	0.64
average path length [95% confidence interval]	*	422 [55–958]	451 [56–1,253]	499 [58–1,166]	296 [42–601]
Assortativity	*	0.58	0.78	0.87	0.91
Mean degree [2.5th and 97.5th percentiles]	*	6.99 [1 - 18]	12.01 [1 - 32]	23.92 [3 - 64]	64.55 [9 - 173]
Mean betweenness centrality [2.5th and 97.5th percentiles]	*	1.79x10^-5^ [0 - 5.16x10^-5^]	3.93x10^-5^ [0 - 1.47x10^-4^]	9.44x10^-5^ [0 - 2.99x10^-4^]	8.87x10^-5^ [1.11x10^-13^ - 4.48x10^-4^]

(* not computed because of the small size of the largest connected component).

**Fig 3:**
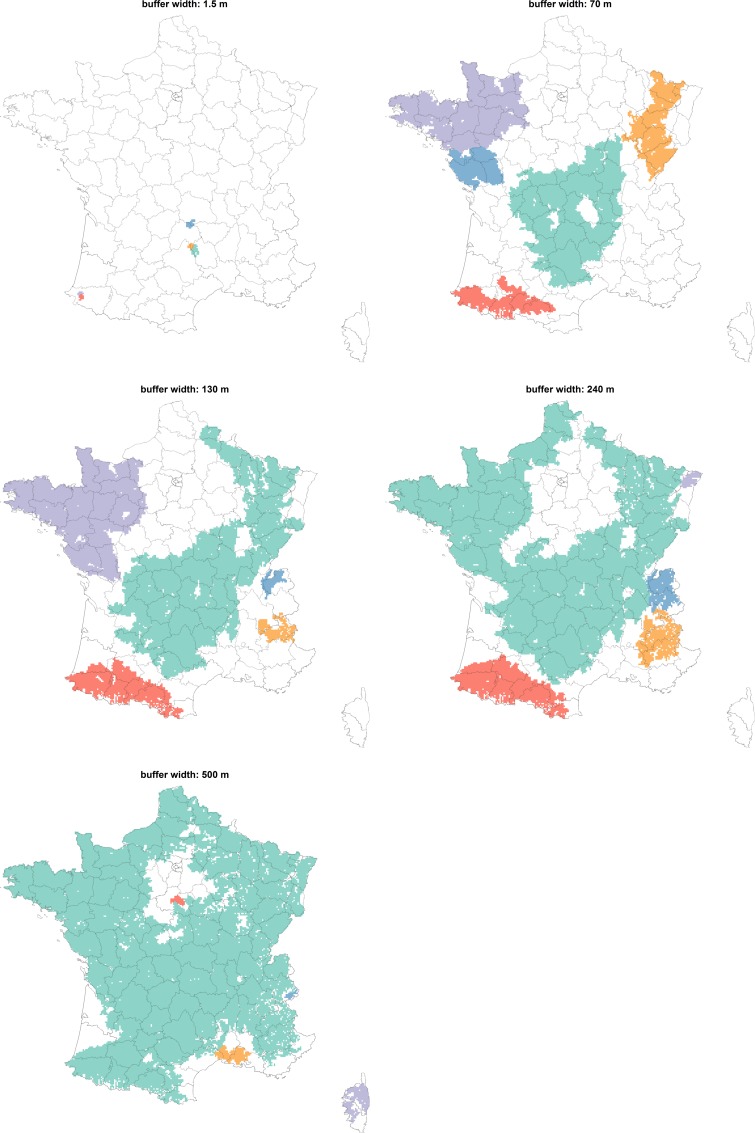
Geographic repartition of the five largest connected components for five pastures networks obtained with increasing buffer widths. Pastures were aggregated using a square grid (cells of 16km^2^). Cells were coloured if at least one pastures belonged to one of the five largest connected components (one colour per connected component).

As the buffer width increased, the connection between the pastures also intensified (**Table 3**). The density and the mean degree were multiplied by 42 between the network obtained with the 1.5-meter buffer width and the network obtained with the 500-meter buffer width (**Fig 2B**). As expected, this increasing connectivity between nodes was mainly local. Indeed, the clustering coefficient doubled between the network obtained with the 1.5 meter buffer width and the network obtained with the 240-meter buffer width, and then reached a plateau around 0.64 (**Fig 2C**). Beginning in the centre (“Massif Central”) and the southern (the Pyrenees) areas of France, the largest connected components progressively grew and merged when the buffer width increased (**Fig 3**).

The clustering coefficients of the pastures networks were several orders of magnitude (more than 20-times) greater than the ones of equivalent random networks (i.e. having the same numbers of nodes and links). Indeed, the clustering coefficients of the pastures networks rose from 0.53 to 0.64 with increasing buffer widths, whereas the clustering coefficients of random networks varied between 2x10^-6^ and 2x10^-5^. The average path length of the pastures networks were more than 50 times larger than for the equivalent random networks (between 296 and 499 versus between 4 and 8, S3 Appendix). The degree distributions of the pastures networks, plotted on a log-log scale, did not show a clear linear trend (S4 Appendix).

To analyse how other indirect contacts between pastures of the same premises did impact the structure of the whole network, links between non-connected pastures of a same premises were added with a probability *p*. For the pastures network obtained with the 1.5-meter buffer width, the largest connected component included 50% of all the French pastures as soon as *p* reached 0.024 (range of 0.022–0.026 in 5 repetitions). This threshold fell below 0.010 for the pastures network obtained using larger buffer widths.

### 3.2. Premises networks analyses and disease implications

**Fig 4:**
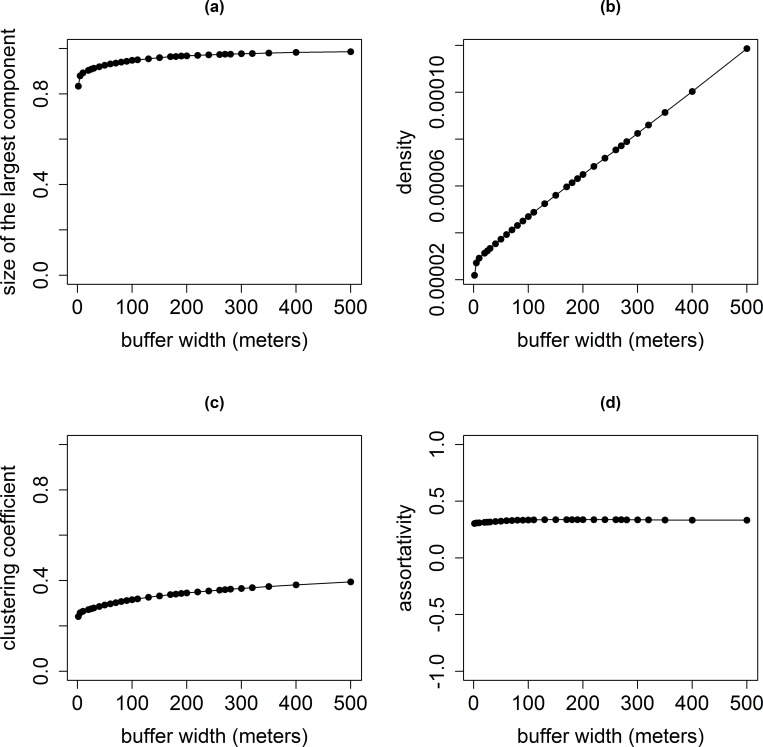
Evolution of indicators for the premises networks according to the buffer width. (a): The proportion of the premises in the largest connected component; (b): the density; (c): the clustering coefficient; (d): the assortativity.

**Fig 5:**
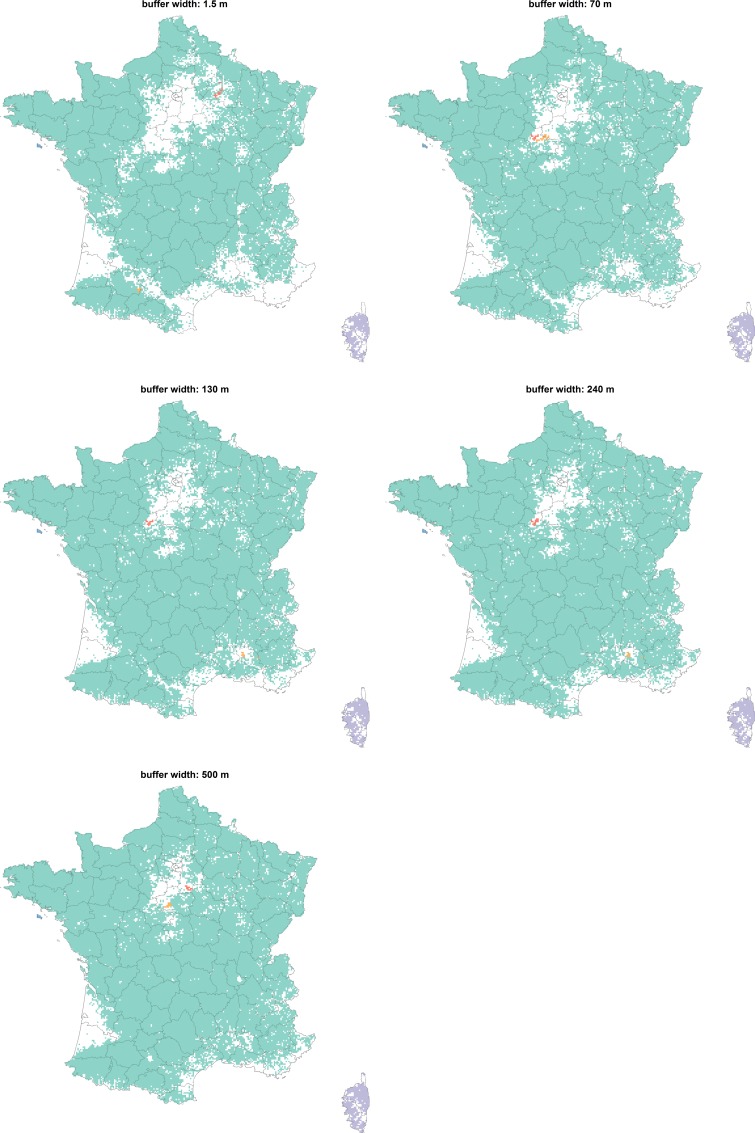
Premises of the five largest connected components for the five networks obtained with increasing buffer widths. Premises were aggregated using a square grid (cells 16km^2^). Cells were coloured if at least one premises belonged to one of the five largest connected components (one colour per connected component).

The premises networks appeared markedly more connected than the pastures networks as the largest connected component of the network obtained with the smallest buffer width (1.5 m) already included more than 80% of the premises (**Fig 4A, Table 4, Fig 5**). This proportion rose to 99% of the premises for the network obtained with the largest buffer width (500 m). The network density linearly rose with the buffer width (slope: 0.18x10^-6^ and 0.05, respectively). The clustering coefficient was multiplied by 1.6 between the networks obtained with the smallest and the largest buffer widths.

**Table 4:** Network indicators describing five premises networks obtained using increasing buffer widths.

Buffer width	1.5 m	70 m	130 m	240 m	500 m
Number of nodes	288,066	288,066	288,066	288,066	288,066
Number of links	910,121	1,712,789	2,176,218	2,984,761	4,924,174
Size of the largest connected component (% of the French premises that owned pastures)	240,349 (83%)	269,738 (94%)	275,177 (96%)	280,006 (97%)	283,983 (99%)
Density (x10^-6^)	21.94	41.28	52.45	71.94	118.68
Clustering coefficient	0.24	0.30	0.33	0.35	0.39
Average path length [95% confidence interval]	16 [7–25]	13 [6–20]	12 [6–18]	11 [5–16]	9 [5–13]
Assortativity	0.30	0.33	0.34	0.34	0.33
Mean degree [2.5th and 97.5th percentiles]	6.32 [0 - 23]	11.89 [0 - 40]	15.11 [1 - 50]	20.72 [1 - 66]	34.19 [3 - 104]
Mean betweenness centrality [2.5th and 97.5th percentiles]	4.11x10^-5^ [0 - 2.74x10^-4^]	3.72x10^-5^ [0 - 2.54x10^-4^]	3.50x10^-5^ [0 – 2.43x10^-4^]	3.22x10^-5^ [0 - 2.21x10^-4^]	2.77x10^-5^ [0 - 1.89x10^-4^]

The clustering coefficients of the premises networks were again several orders of magnitude (more than 20-times) larger than the clustering coefficients of random networks having the same numbers of nodes and links. The clustering coefficients of the premises networks rose from 0.24 to 0.39 with increasing buffer widths, whereas the ones of the random networks ranged between 2.8x10^-5^ and 1.2x10^-4^ (S3 Appendix). The average path length for each premises network and the equivalent random networks were similar (from 9 to 16 for the premises network and from 4 to 7 for the equivalent random networks). The log-log plots of the degree distributions did not show a linear shape (S5 Appendix).

**Fig 6:**
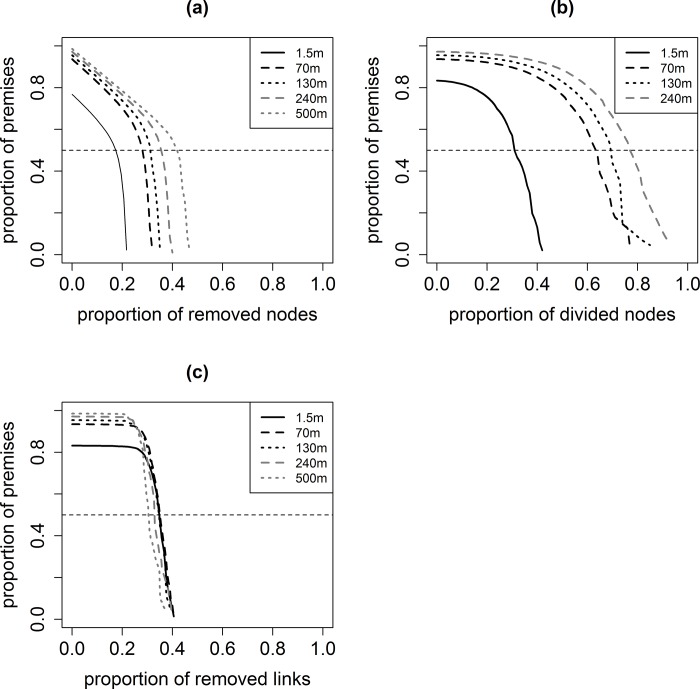
Evolution of the proportion of premises within the largest connected component for the three percolation analysis for the networks obtained with several buffer width. (a) Strict biosecurity: proportion of nodes removed; (b) within-premises biosecurity: proportion of nodes divided; (c) between-premises biosecurity: proportion of links removed; horizontal dotted line: 0.5 (i.e. the threshold used to define the disappearance of the giant component).

The smallest percolation thresholds were obtained with the node removal procedure (i.e. strict biosecurity): depending on the buffer width, between 17% and 42% of the nodes had to be removed to maintain the size of the largest connected component below 50% of the network size (see **Table 5**, and **Fig 6**). For the within-premises biosecurity measures, the percolation threshold was 31%, 63%, 69% and 79% for the premises network obtained with the 1.5-, 70-, 130- and 240-meter buffer width respectively. No percolation threshold could be obtained for the last buffer width (500 m): the simulated implementation of within-premises biosecurity measures did not allow reducing the size of the largest connected component below 50% of the network size. This is explained by the fact that (i) the implementation of within-premises biosecurity measures progressively transformed the premises network into the pastures network, and (ii) the size of the largest component of this latter was already >50% of the nodes. For the between-premises biosecurity measures, the percolation threshold was always between 30% and 35%, whatever the buffer width. Except for the between-premises biosecurity measures, the buffer width had a marked effect on the percolation threshold, especially between the premises networks obtained with the 1.5-meter and the 70-meter buffer widths when implementing within-premises biosecurity. Differences became less marked for higher buffer widths (**Table 5**).

**Table 5:** Percolation thresholds in the premises networks, and population-level efficacy of biosecurity measures.

Buffer width	1.5 m	70 m	130 m	240 m	500 m
Number of nodes	288,066	288,066	288,066	288,066	288,066
Number of links	910,121	1,712,789	2,176,218	2,984,761	4,924,174
Strict biosecurity	50,122 (17%)	80,732 (28%)	89,912 (31%)	102,263 (35%)	120,446 (42%)
Within-premises biosecurity	89,175 (31%)	182,441 (63%)	199,637 (69%)	221,760 (79%)	NT
Between-premises biosecurity	316,239 (35%)	599,738 (35%)	753,994 (35%)	979,594 (33%)	1,499,845 (30%)

Percolation threshold: number (brackets: proportion of initial network size) of removed (or transformed) nodes/links necessary to reduce the size of the largest connected component to <50% of the network size. Strict biosecurity: number of nodes removed; within-premises biosecurity: number of nodes transformed; between-premises biosecurity: number of links removed, NT: no percolation threshold.

## 4. Discussion

The aim of this study was to analyse, at the country level, the structural risk of disease spread between animals located on neighbouring pastures. Based on GIS data, the spatial organization of pastures was represented using networks. These networks were analysed to address two questions: (i) is the spatial configuration of pastures favourable to the spread of infectious diseases in France? (ii) if yes, which biosecurity measures would allow decreasing this vulnerability, and to what extent would they need to be adopted by breeders?

Several buffer widths were used to define connected pastures: a link was created when the distance between pastures boundaries was below a predefined threshold. A more accurate representation would have associated a weight to each link, this weight decreasing with the distance between pastures (according to some spatial kernel). Although it would have been more realistic for the largest buffer widths, the simple approach we adopted here allowed using the same representation of the transmission risk for each buffer width (thus addressing both directly- and indirectly-transmitted diseases) without having to specify additional parameters for the kernel shape. The population level efficacy of three levels of biosecurity measures (strict biosecurity, within-premises biosecurity and between-premises biosecurity) was also assessed through percolation analyses, assuming a 100% efficacy of these measures at the premises level. This is an optimistic scenario, and taking into account a non-perfect efficacy of biosecurity measures would have resulted in higher percolation thresholds (i.e. to be effective at the population level, a non-perfect biosecurity measure would need to be adopted by more breeders).

The 1.5-meter buffer width represented the transmission risk through direct contacts (nose-to-nose contacts over the fences), while taking into account the geographic accuracy of pastures borders reported by breeders (a few meters). For this width, the pastures network was strongly fragmented with a largest connected component including less than 1% of the 3,159,787 French pastures. However, this fragmentation disappeared with larger buffer widths: the wider the buffer was, the more connected the pastures network became. More than 75% and nearly all (97%) of the French pastures were connected with the 240-meter buffer width and a 500-meter buffer width, respectively. These results suggest that a directly-transmitted disease should not be able to spread through contacts between animals over the fences (pastures network with a 1.5 m buffer width), whereas a vector-borne disease would spread quickly (pastures network with a 500 m buffer width). However, these results only hold if breeders move neither animals nor farm equipment from one of their pastures to another one. Randomly connecting pastures that belong to the same premises allowed representing such movements. A connection probability between 0.02 and 0.03 was enough to connect 50% of the French pastures in the network obtained with the 1.5-meter buffer width. This probability fell below 0.01 for a 70-meter buffer width. This shows that despite its fragmentation, a directly-transmitted disease could quickly spread in the pastures network, unless distinct pastures are managed separately by breeders.

Contrary to the pastures network, the premises network was highly connected with a largest connected component that included 83% of the 288,066 French premises in the network obtained with a 1.5-meter buffer width (99% for a 500-meter buffer width). This high connectivity level could be slightly underestimated, as our dataset did not allow identifying collective pastures, shared by several premises, which would increase the connectivity between premises. Such collective pastures are however rare in France (<1,500 reported in 2010, source: agricultural census).

Neither the pastures nor the premises networks did show the properties of scale-free networks. The degree distributions on a log-log scale did not appear to be linear, and the networks were assortative, contrary to scale-free networks, which are disassortative, with highly connected nodes (so-called “hubs”) being linked to weakly connected ones [21,24]. This was an expected result for the pastures networks due to the spatial constraints, but properties of scale-free networks could have been (at least theoretically) possible for the premises networks, as some premises owning many pastures in various locations could have played the role of hubs. The spatial nature of pastures networks also explains the fact that these networks did not show the properties of small-world networks. Although, as in small-world networks, the clustering coefficient was several orders of magnitude higher than the one of equivalent random networks (i.e. having the same number of nodes and links), the average path length was also much higher than the one of equivalent random networks, whereas it is not the case in typical small-world networks [37]. Premises networks appeared to be closer to small-world networks than pastures networks: the clustering coefficient was again high (several orders of magnitude higher than the one of equivalent random networks), but the average path length had the same order of magnitude than the one of equivalent random networks. In these premises networks, the combination of spatial constraints (inducing high clustering) with the existence of premises owning many pastures in several locations (thus connecting distant areas of the network) could explain these small-world properties.

Three types of biosecurity measures were addressed using three percolations analyses that showed the difficulty to prevent the spread of diseases on pastures. The first percolation analysis used a node removal procedure that modelled a strict biosecurity measure such as the confinement of animals inside buildings. To stop the spread of a directly-transmitted pathogen, the biosecurity measure had to be implemented in more than 17% of the French premises. This proportion rose above 30% for networks obtained using buffer widths >130 meters, which may be relevant for a pathogen with air-borne transmission. Within-premises biosecurity measures such as the standstill of animal movements between pastures may also allow limiting the spread of diseases. However, there was no percolation threshold for the corresponding percolation analysis for the network obtained with the 500-meter buffer width. Hence, within-premises biosecurity measures alone did not allow preventing the spread of a vector-borne disease. The last type of studied biosecurity measures was the between-premises biosecurity measures such as the strengthening of fences, or grazing animals on pastures without neighbouring pastures. Interestingly, whatever the buffer width, the percolation threshold was between 30% and 35%. At the percolation threshold, the number of removed links increased with the buffer width, but also the total number of links. The proportion of removed links thus remained stable.

Therefore, it appeared possible to decrease the structural risk of the French premises network to the spread of an infectious disease transmitted by contacts on pastures. However, whatever the type of biosecurity measures, they have to be applied by a large number of breeders to become effective at the population level.

The relationship between the size of the largest connected component and the buffer width was irregular with plateaus separating sharp increases. This shape can be explained by the co-occurrence of two types of processes (**Fig 2A**). Increasing the buffer width first induces a slow increase of the size of existing connected components, corresponding to the plateaus. However for several buffer widths, due to the spatial nature of the network, connected components merge, inducing sharp increases of the size of the largest connected component.

Contrary to the “pastures component” of disease transmission risk that is, as far as we know, analysed here for the first time, the “animal trade” component has been studied in several countries using network analysis methods. Networks were considered as static in the earliest studies [33,34,41]. However, as shown by Vernon and Keeling [42], the temporal dynamics of cattle trade biased the analyses performed with the static approximation. Therefore, the temporal component of these networks has to be taken into account [25,43]. Studies of cattle movement networks now use methods dedicated to temporal networks [22,24,44]. Indeed, networks of animal trade model contact events (due to the movement of one or several animals) between premises: the temporal sequence of these events thus strongly impacts the overall disease spreading risk. Similarly to cattle movement networks, the pastures network analysed here also has a temporal dimension: animals usually stay on a given pasture during several weeks or months (means of 130 days [10–225] in 10 herds in Côte d’Or—Malika Bouchez-Zacria personal communication), and neighbouring pastures are not necessarily used at the same time. However, contrary to animal trade networks, pastures networks do not model contact events but contact periods between premises. Thus, using a static analysis appears to be a more valid approximation for the pastures network than for the animal trade network. Nevertheless, if temporal data on pastures usage would become available, it would be interesting to implement temporal network methods to analyse the pastures network. Furthermore, the knowledge of temporal data for the pastures network would allow quantifying the goodness of the static approximation with the causal fidelity introduced by Lentz *et al*. [45].

It is interesting to compare the cattle trade network and the pastures network for the vulnerability to infectious disease spread. Rautureau *et al* [21] showed that less than 1% of the premises (mostly markets or dealers) had to be closed (or submitted to confinement measures) to stop the spread of a disease through the French cattle trade network. It means that the closure of a small number of targeted premises was very effective to prevent the spread of a disease. Consistent results were obtained in other countries and/or for other species, like the cattle trade network in Italy [25,33], the pig trade network in Germany [26,46] or the sheep trade network in Great Britain [27]. On the contrary, the network of pastures is much more vulnerable to the spread of infectious diseases because of the high level of connection between the premises and the inherent difficulty to stop the spread of a disease on this network, using biosecurity measures. This difference seems mainly due to the structure of the network. Contrary to the pastures network, the cattle trade network shows scale-free properties [21,22]. In scale-free networks, the most highly connected nodes (or hubs) play a strong role in the spread of a disease. Thus, biosecurity measures that target these nodes are very effective [47]. The pastures network is a spatial network. This spatiality induces constraints on the network structure, because of the bi-dimensional organisation of the pastures [48].

Besides, for a given disease, the “pastures component” is only one part of the global between-premises transmission risk, which also includes the “cattle trade” component. Although it is not currently possible in France, due to the mandatory anonymization of pastures data, it would be interesting in the future to combine both components in order to analyse the global transmission risk. Furthermore, as diseases do not know boundaries (as proved by the Bluetongue epidemic in 2006), it would thus be relevant to study both components at the European scale.

## 5. Conclusion

The spatial organization of pastures was represented using networks, and network analysis methods were applied on pastures networks and premises networks to analyse their vulnerability to the spread of infectious diseases. By modifying the threshold for the definition of “pastures in contact”, it was possible to study the transmission of several types of diseases on these networks. We showed that the pastures network was rather fragmented, especially in the case of a disease transmitted through direct contacts. However, most of the premises own several pastures, and at the premises scale, the network was much more connected with a largest connected component that included more than 83% of the premises. This indicates a marked vulnerability of this network to the spread of infectious diseases in France. Finally, percolations analyses showed the difficulty to limit the spread of diseases between pastures in France. A large adoption of biosecurity measures by breeders is therefore necessary to reduce the vulnerability of the network to the spread of directly-transmitted diseases.

## Supporting Information

S1 Appendix Comparison of the percolation results according to the selection method of the removed nodes for the premises network obtained with the 1.5m buffer width. Random procedure, targeted procedure according to the degree, targeted procedure according to the betweenness centrality.
(PDF)

S2 Appendix Comparison of the percolation results according to the number of nodes removed at each step for the premises network obtained with the 1.5m buffer width. 0.1%, 0.5%, 1%, 5% or 10% of the initial network size
(PDF)

S3 Appendix Comparison of the clustering coefficient and the average path length between the observed networks and random networks (same number of nodes and links). (PDF)

S4 Appendix Degree distributions for the pastures networks obtained with several buffer widths (1.5, 70, 130, 240 and 500 meters). Graph with log-log scale.
(TIFF)

S5 Appendix Degree distributions for the premises networks obtained with several buffer widths (1.5, 70, 130, 240 and 500 meters). Graph with log-log scale.
(TIFF)
