# Supplementary material for: Analysis of the Spatial Organization of Pastures as a Contact Network, Implications for Potential Disease Spread and Biosecurity in Livestock, France, 2010
Source: PLoS One. 2017 Jan 6;12(1):e0169881. doi: 10.1371/journal.pone.0169881 (PMC5218577; doi:10.1371/journal.pone.0169881)
Supplement: S1 Appendix — Random procedure, targeted procedure according to the degree, targeted procedure according to the betweenness centrality. (PDF) [file pone.0169881.s001.pdf]

Selection methods of the percolation analyses

The percolation analyses were based upon the following procedure:

(i) select 1% of nodes,

(ii) remove the selected nodes,

(iii) calculate the size of the largest connected component,

(iv) if this size is <50% of the nodes (i.e. if the giant connected component has disappeared) stop, else go to step (i).

At step (i), the selection of nodes/links in the network can be random or targeted, based on node- of link-level indicators: the degree (selected nodes are the ones with the highest degree) or the betweenness centrality (selected nodes or links are the ones with the highest betweenness centrality). Because of the large size of the studied networks and to keep computation time reasonable, the betweenness centrality was estimated based on shortest paths of length ≤3. Three selection methods (random, targeted according to the degree, targeted according to the betweenness centrality) were first compared to identify the most effective one, using node removal: in step (i) of the above procedure, the selected elements were nodes, and the change applied in step (ii) consisted in removing the selected nodes.

The comparison showed that the size of the largest connected component decreased faster with the targeted selection procedures than with the random selection procedure: the percolation threshold was 25% of the initial network size for the random selection procedure, 18% for the degree-based selection procedure, and 17% for the betweenness-based selection procedure (**Fig.**). The betweenness-based selection procedure was thus the most effective of the three selection methods.

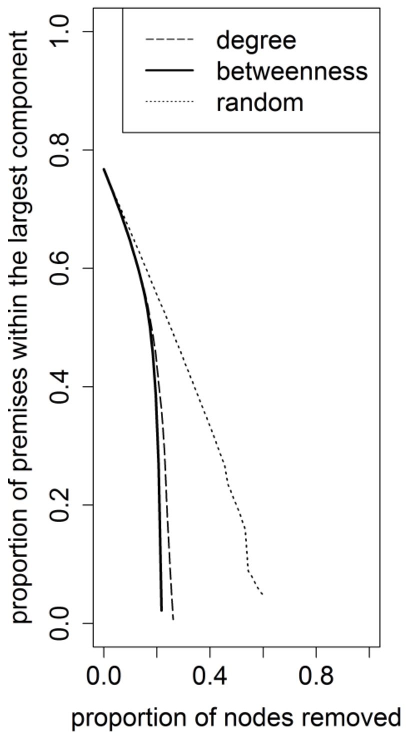

Fig. Comparison of percolation results according to the selection method of the removed nodes. Random procedure, targeted procedure according to the degree, and targeted procedure according to the betweenness centrality; with the premises network obtained with the 1.5m buffer width.
