# Supplementary material for: Analysis of the Spatial Organization of Pastures as a Contact Network, Implications for Potential Disease Spread and Biosecurity in Livestock, France, 2010
Source: PLoS One. 2017 Jan 6;12(1):e0169881. doi: 10.1371/journal.pone.0169881 (PMC5218577; doi:10.1371/journal.pone.0169881)
Supplement: S2 Appendix — 0.1%, 0.5%, 1%, 5% or 10% of the initial network size (PDF) [file pone.0169881.s002.pdf]

Proportion of nodes selected at each step of the percolation analyses

The percolation analyses were based upon the following general procedure:

(i) select a proportion *q* of nodes with the highest betweenness centrality,

(ii) removed the selected nodes,

(iii) calculate the size of the largest connected component,

(iv) if this size is <50% of the nodes (i.e. if the giant connected component has disappeared) stop, else go to step (i).

Using the targeted selection based on the betweenness centrality, the node percolation analysis was repeated with different proportions *q* of nodes removed at each step. The number *q* was expressed as a percentage of the initial network size and five values were compared (0.1%, 0.5%, 1%, 5% and 10% of the initial network size) to determine the value of *q* that allowed obtaining a good precision of percolation threshold estimate, while keeping computation time reasonable.

The proportion *q* of nodes removed at each time step did slightly influence the percolation analysis results (**Fig.**). Indeed the percolation threshold was 17% of the initial network size for *q*=0.1%, 17% for *q*=1%, and 15% for *q*=10%.

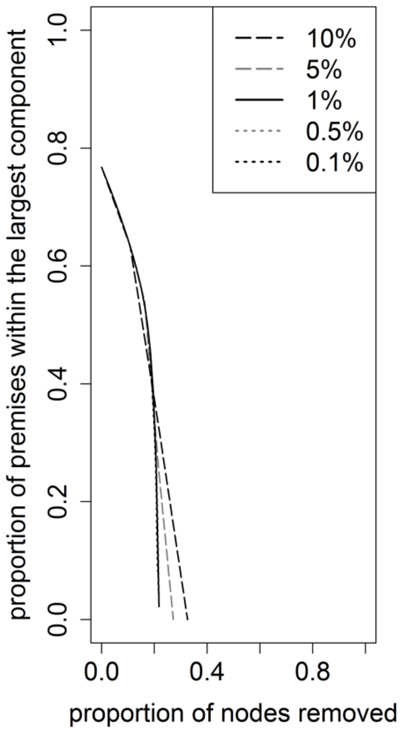

Fig. Comparison of the percolation results according to the number of nodes randomly removed at each step. 0.1%, 0.5%, 1%, 5% or 10% of the initial network size; premises network obtained with the 1.5m buffer width.
