# Supplementary material for: Analysis of the Spatial Organization of Pastures as a Contact Network, Implications for Potential Disease Spread and Biosecurity in Livestock, France, 2010
Source: PLoS One. 2017 Jan 6;12(1):e0169881. doi: 10.1371/journal.pone.0169881 (PMC5218577; doi:10.1371/journal.pone.0169881)
Supplement: S3 Appendix — (PDF) [file pone.0169881.s003.pdf]

Comparison with random networks

In order to assess if the pastures or the premises networks displayed small-world properties, random networks were generated for each observed network. The random networks had the same numbers of nodes and links as the corresponding observed network. For each network (observed and random), the clustering coefficient and the average path length were estimated (Tables 1 and 2). The average path lengths were estimated with a 10,000-node sample due to computational time.

No results are presented for the pastures network obtained with a 1.5-meter buffer width because of the small size of the largest component (< 1% of all nodes).

**Buffer width**	**70 m**	**130 m**	**240 m**	**500 m**
**Number of nodes**	3,159,787	3,159,787	3,159,787	3,159,787
**Number of links**	11,049,232	18,978,231	37,794,387	101,984,579
**Clustering coefficient**				
Observed network	0.53	0.59	0.62	0.64
Random network	2.5×10^{-6}	3.9×10^{-6}	7.4×10^{-6}	2.0×10^{-5}
**Average path length [95% confidence interval]**				
Observed network	422 [55 – 958]	451 [56 – 1,253]	499 [58 – 1,166]	296 [42 – 601]
Random network	8 [6 – 9]	6 [5 – 7]	5 [4 – 6]	4 [3 – 5]

Table 1. Comparison between pastures networks and equivalent random networks.

**Buffer width**	**1.5 m**	**70 m**	**130 m**	**240 m**	**500 m**
**Number of nodes**	288,066	288,066	288,066	288,066	288,066
**Number of links**	910,121	1,712,789	2,176,218	2,984,761	4,924,174
**Clustering coefficient**					
Observed network	0.24	0.30	0.33	0.35	0.39
Random network	2.8×10^{-5}	4.2×10^{-5}	5.5×10^{-5}	7.6×10^{-5}	1.2×10^{-4}
**Average path length [95% confidence interval]**					
Observed network	16 [7 – 25]	13 [6 – 20]	12 [6 – 18]	11 [5 – 16]	9 [5 – 13]
Random network	7 [5 – 8]	5 [4 – 6]	5 [4 – 6]	5 [3 – 5]	4 [3 – 5]

Table 2. Comparison between premises networks and equivalent random networks.
