# Supplementary figures and images for: Analysis of the Spatial Organization of Pastures as a Contact Network, Implications for Potential Disease Spread and Biosecurity in Livestock, France, 2010
Source: PLoS One. 2017 Jan 6;12(1):e0169881. doi: 10.1371/journal.pone.0169881 (PMC5218577; doi:10.1371/journal.pone.0169881)

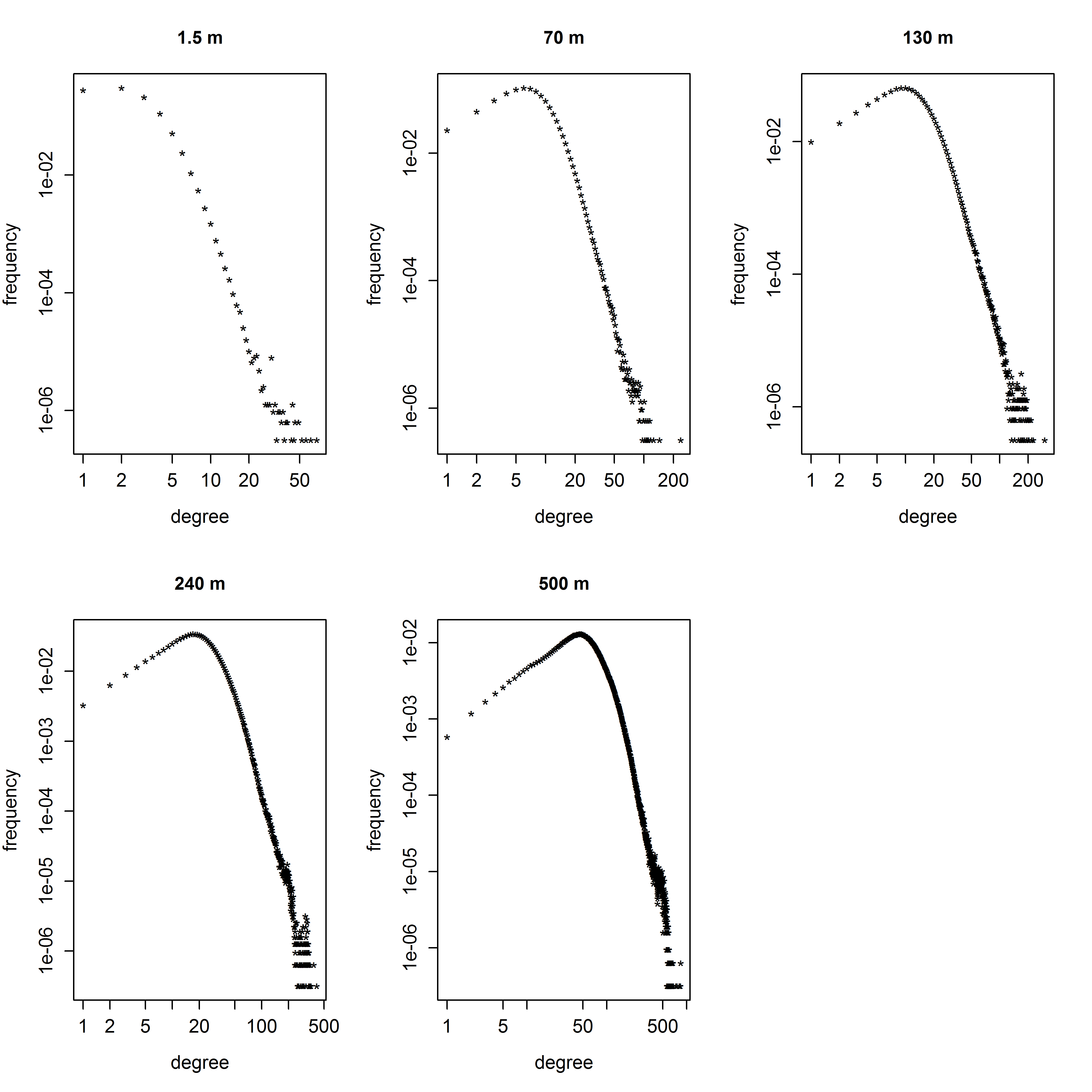

Supplement: S4 Appendix — Graph with log-log scale. (TIFF) [file pone.0169881.s004.tiff]

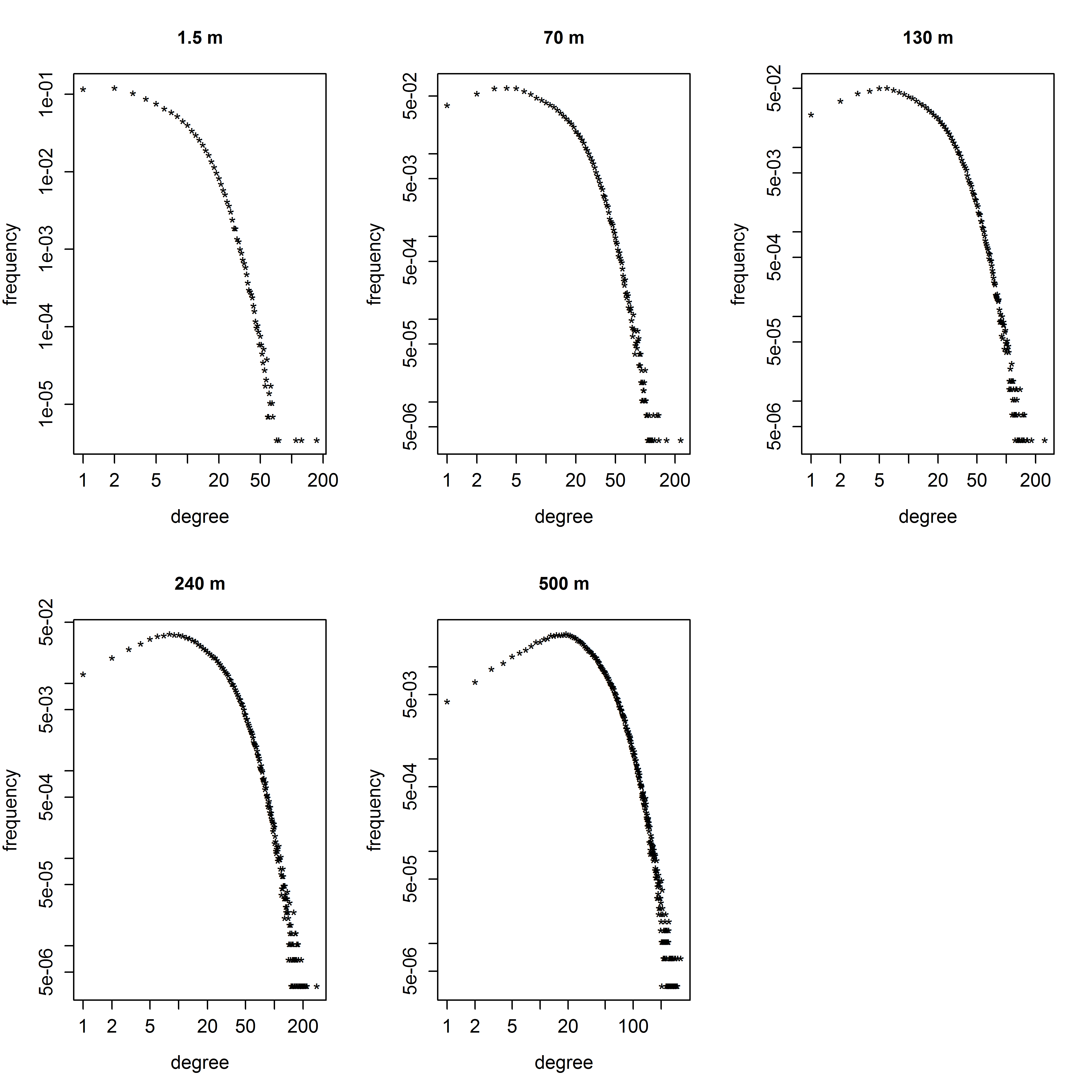

Supplement: S5 Appendix — Graph with log-log scale. (TIFF) [file pone.0169881.s005.tiff]
